# Astaxanthin inhibits aldose reductase activity in *Psammomys obesus*, a model of type 2 diabetes and diabetic retinopathy

**DOI:** 10.1002/fsn3.1259

**Published:** 2019-11-12

**Authors:** Maha Benlarbi‐Ben Khedher, Khouloud Hajri, Ahmed Dellaa, Basma Baccouche, Imane Hammoum, Nourhene Boudhrioua‐Mihoubi, Wissal Dhifi, Rafika Ben Chaouacha‐Chekir

**Affiliations:** ^1^ Laboratory of Physiopathology, Food and Biomolecules (PAB): LR17ES03 The High Institute of Biotechnology of Sidi Thabet (ISBST) University of Manouba (UMA) Sidi Thabet Tunisia

**Keywords:** aldose reductase, astaxanthin, diabetic retinopathy, *Psammomys obesus*

## Abstract

Astaxanthin (ATX) is a marine carotenoid known for its powerful antioxidant and neuroprotective properties. In this study, we investigated the in vitro and in vivo potential inhibitory effect of ATX on the aldose reductase (AR) activity, a key enzyme in the polyol pathway responsible for the pathogenesis of diabetic complications including diabetic retinopathy (DR). The gerbil *Psammomys obesus* (*P. ob.*), an animal model for type 2 diabetes and DR has been used. The erythrocyte and retinal AR activity of *P. ob.* individuals were, respectively, assessed monthly and at the 7th month during a 7‐month hypercaloric diet (HD) using a NADPH oxidation method. Meanwhile, the body weight and blood glucose of the gerbils were monitored. After 7 months, *P. ob.* individuals were fed with ATX (4.8 mg/kg of body weight) once a day for 1 week. The results showed that the HD‐fed animals developed significant obesity and hyperglycemia in comparison with controls. Erythrocyte AR activity showed a progressive and significant increase in the HD‐fed group compared with controls. Retinal AR activity was higher in the 7‐month HD‐fed group compared with controls. Erythrocyte AR activity was markedly decreased after ATX‐treatment in vitro and in vivo. These findings suggested that ATX inhibited the erythrocyte AR activity and could be used for DR prevention and/or early treatment.

## Graphical abstract

We investigated the in vitro and in vivo potential inhibitory effect of astaxanthin on the aldose reductase (AR) activity, a key enzyme in the polyol pathway responsible for the pathogenesis of diabetic complications including diabetic retinopathy (DR) in *Psammomys obesus*. Our results showed that astaxanthin may prevent and treat diabetes complications.

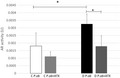

## 1 INTRODUCTION

Rapid urbanisation and life style change accelerated the epidemic expansion of diabetes (Hu, 2011). Hyperglycemia, obesity, and insulin resistance are associated with type 2 diabetes and metabolic disorders. The development of diabetic complications is linked to diabetes duration in patients (Ahmed, Khalil, & Al‐Qahtani, 2016; Cai, Wang, & Ji, 2006; Hussain, Qamar, Iqbal, Ahmad, & Ullah, 2013; Klein, Klein, Moss, Davis, & DeMets, 1984).

Diabetic retinopathy (DR) is a visual deficiency that is one of the major complications of long‐term diabetes affecting approximately 4.2 million people worldwide and representing a leading cause of blindness. It is a microvascular retinal disease characterized by vascular occlusions, increased capillary permeability, loss of pericytes, microaneurism, intraretinal hemorragies, macular edema, and neovascularization (Hendrick, Gibson, & Kulshreshtha, 2015).

Previous studies indicated that several biochemical pathways have been proposed to explain the effect of hyperglycemia in the development of microvascular complications including the activation of the polyol pathway and of the protein kinase C, the increased formation of advanced glycation end products and oxidative stress (Brownlee, 2005; Geraldes & King, 2010; Giacco & Brownlee, 2010)**.** Among these, the polyol pathway has been extensively studied involving aldose reductase (AR), the first and rate‐limiting enzyme in this pathway belonging to the aldo‐keto reductase superfamily. It reduces the excess of glucose to sorbitol using nicotinamide adenine dinucleotide phosphate (NADPH) as cofactor. Sorbitol is then metabolized to fructose by the sorbitol dehydrogenase (Das‐Evcimen et al., 2012). Previous researches demonstrated that activation of polyol pathway induced intracellular hyperosmolarity throughout the accumulation of sorbitol (Lorenzi, 2007), oxidative stress (Giacco & Brownlee, 2010)**,** alteration of the ratio of NADPH/NADP (Brownlee, 2005) and NADH/NAD (Lorenzi, 2007), and myo‐inositol depletion (Chung & Chung, 2005).

*Psammomys obesus* (*P. ob.*), a semi‐desertic rodent, is an extensively used model for nutritionally induced diabetes and metabolic syndromes (Leibowitz et al., 2001; Marquie, Duhault, & Jacotot, 1984). When subjected to a hyper caloric diet (HD), it has been shown that this rodent develops type 2 diabetes and DR with similar functional and structural alterations to that observed in humans (Saïdi, Mbarek, Chaouacha‐Chekir, & Hicks, 2011; Saïdi, Mbarek, Omri, et al., 2011). This suggests that *P. ob.* can be used as a valuable model for screening new therapeutic strategies for DR.

Aldose reductase (AR) is a target for the treatment of diabetic complications. Considerable effort has been devoted to the study of several AR inhibitors extracted from the biomass and which have shown promising effect by preventing and slowing the progression of DR (Akileshwari et al., 2012; Duan, Huang, Li, & Tang, 2013; Kim, Kim, Sohn, Lee, & Kim, 2011; Liu et al., 2008).

Astaxanthin (ATX) is a potent natural antioxidant belonging to the xanthophyll carotenoid family occurring in crustaceans, salmons, and crabs. It is used as a dietary supplement to promote health condition. ATX has been investigated for its anti‐inflammatory (Yang, Kim, & Lee, 2013), antitumoral (Zhu, 2013), and antiaging potentials (Kidd, 2011). In diabetic studies, ATX decreased blood glucose level in diabetic mice (db/db) (Uchiyama et al., 2002), inhibited lipid peroxidation (Marin, Bolin, Macedo, Sampaio, & Otton, 2011), and decrease oxidative stress in alloxan diabetic rats (Wang, Chen, & Lu, 2012). As reported by Baccouche et al. (2017) and Baccouche, Benlarbi, Barber, and Ben Chaouacha‐Chekir (2018), respectively, in vitro and in vivo, ATX exerted neuroprotection against high glucose‐induced damage on *P. ob.* retinal cells. However, there have been no reports studying the inhibitory effect of ATX on AR activity. Based on these observations, the aim of this paper was to determine erythrocyte AR activity of *P. ob.* exposed to HD and evaluate the potential inhibitory effect of ATX on this activity both in vitro and in vivo.

## 2 MATERIALS AND METHODS

### 2.1 Animals

Experiments were performed in young adult *P. ob.* captured from the southern region of Tunisia (Bouhedma Park) with the authorization of Tunisian Agriculture Ministry (number of approval: 2012‐2016/2214‐1693). Gerbils were transferred to animal facilities and kept in standard laboratory conditions: 12 hr light and dark cycle, a constant temperature (25 ± 2°C), relative humidity was maintained at 70 ± 10% with free access of water and food. Animal experimentation was conducted in accordance with the ethical guidelines of the Pasteur institute ethics committee of Tunisia (number of approval: 2016/11/E/ISBST/V1). Animals were used and handled according to the principles of the Association for Research and Vision Ophthalmology (ARVO) Statement for the Use of Animals in Ophthalmology and Vision Research.

### 2.2 Diabetes induction

Animals were acclimated for a couple of weeks and received a natural vegetable diet, that is, halophilic plants rich in water and mineral salts (0.4 Kcal/g wet weights). Data presented in this study came from two independent field excursions. Animals were divided randomly into the two following groups: a control group (*n* = 7) was fed with only halophilic plants, and a HD group (*n* = 14) received a standard laboratory rat chow (4 kcal/kg). Both groups were followed up for 7 months with measurements of body weight (every week) and plasma glucose (once a month). The animals were considered diabetic when their blood glucose levels >200 mg/dl.

### 2.3 Aldose reductase assay

#### 2.3.1 Preparation of blood homogenate

Blood was collected from the infraorbital sinus of rats into heparinized tubes. Red blood cells (RBC) were separated by centrifugation at 5,000 *g* for 10 min and transferred immediately for analysis. 10 µl of RBC freshly collected from control *P. ob.* (*n* = 4) and diabetic *P. ob.* (*n* = 4) was added to 90 µl of sodium phosphate buffer (50 mM; pH = 7.4) containing 150 mM NaCl. The suspension was lysed by repeated freezing and thawing for three cycles (Reddy et al., 2008). Erythrocyte AR activity was followed up from the fourth month to the seventh month of hypercaloric diet (4th, 5th, 6th, and 7th).

#### 2.3.2 Preparation of retina homogenate

Animals were humanely sacrificed, euthanized by CO2 inhalation and then decapitated. Eyes were immediately enucleated, rinsed with alcohol and placed into binocular dissecting microscope. Lens and cornea were removed after cutting the anterior segment via an incision in the pars plana. The neural retina was gently isolated from the pigment epithelium and homogenated in 1 ml of potassium phosphate buffer (50 mM, pH = 6.2). The homogenate was centrifuged at 15,000 r/min for 30 min, and the supernatant containing the enzyme fraction was kept in −20°C.

#### 2.3.3 Aldose reductase activity assay

Aldose reductase activity was measured at 340 nm using a spectrophotometer and in the presence of D, L‐glyceraldehyde, a substrate of the enzyme. The assay mixture of 1 ml contained potassium phosphate buffer (50 mM; pH = 6.2), 0.4 mM lithium sulfate, 5 mM mercapthoethanol, 10 mMglyceraldehyde, 10 mMNADPH, and the enzyme fractions preparations from erythrocytes or retina (Duan et al., 2013). The reaction was initiated by the addition of NADPH. The change in the absorbance due to NADPH oxidation was followed. Readings were taken at t1 = 0 min and t2 = 5 min. AR activity unit (U) was defined by the absorbance decrease in the assay mixture per min.

### 2.4 In vitro astaxanthin effect on AR activity

To evaluate the effect of ATX i*n vitro* on AR activity, blood was collected from the control group (*n* = 4) and the 7‐month HD‐fed *P. ob.* group (*n* = 4) then processed as described previously. Measurement of AR activity was performed with or without 50 µg/ml of ATX (Sigma Aldrich, SML 0982) in the assay mixture dissolved in dimethyl sulfoxide (DMSO).

### 2.5 In vivo astaxanthin effect on AR activity

After 7 months of HD, *P. ob.* (*n* = 4) were fed 4.8 mg ATX per kg of their body weight during 7 days. To evaluate the effect of ATX on the AR activity of diabetic *P. ob*., blood was collected and AR activity was measured as described previously and compared with that of diabetic *P. ob.* untreated with ATX (*n* = 4) and to that of a control group (*n* = 4).

### 2.6 Statistical analysis

The results are presented as mean ± standard deviation. A *p* value <.05 was considered statistically significant. Significance between 2 groups was determined by using two factors ANOVA for changes in body weight, blood glucose levels and the evolution of AR activity. Independent sample Student *t* test was used for the measurement of AR activity in retina. One way ANOVA followed by Tukey's post hoc test was used for the measurement of erythrocyte AR in vitro and in vivo. The tests were performed using the SPSS program (version 17).

## 3 RESULTS AND DISCUSSIONS

### 3.1 Body weight change

**Figure 1:**
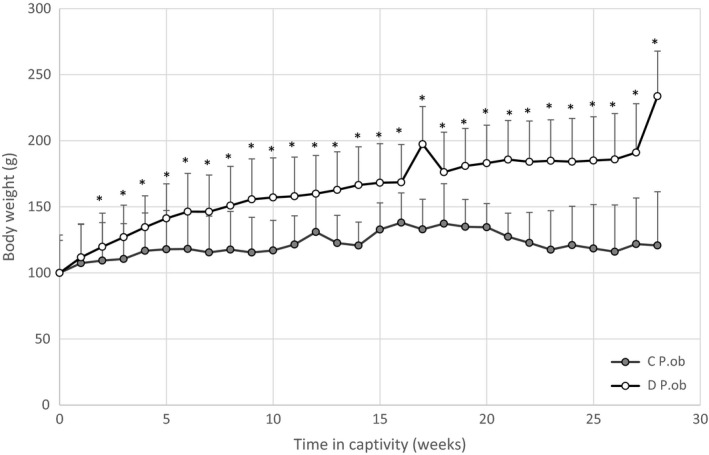
Body weight evolution of *P. ob.* individuals during 7 months of captivity fed with hypercaloric diet. Control (C) *P. ob.* (*n* = 9) weight remained stable through the entire period. Diabetic (D) *P. ob.* (*n* = 14) showed a weight gain under hypercaloric conditions. Data expressed as mean ± standard deviation. (*): designates significant difference in comparison with C group (*p* < .05)

The weight change, determined as a percentage of initial weight of both groups, is shown in Figure 1. These results showed that the weight of the *P. ob.* individuals of the control group did not change significantly throughout the 7‐month experimentation. Indeed, they maintained a roughly stable weight with an increase of approximately 38.06%, probably due to their condition of life in captivity (lack of activity) and/ or to a normal evolution of their weight according to age. However, the *P. ob.* individuals fed with HD developed a progressive increase of their body weight starting from the 2nd week of treatment with a significant gain (233.71%±34.13) of their body weight after 7 months (*p* < .05), in comparison with controls.

### 3.2 Evolution of blood glucose level

**Figure 2:**
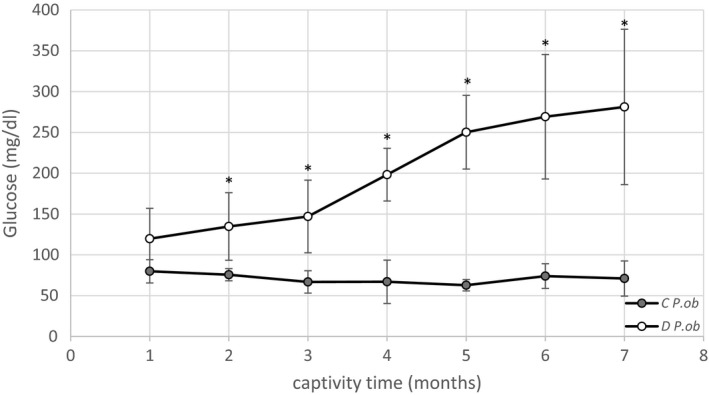
Blood glucose level evolution of *P. ob.* individuals during 7 months of captivity. Control (C) *P. ob.* (*n* = 6) showed a stable blood glucose level along the period of captivity. Diabetic (D) *P. ob.* (*n* = 4) showed a highly significant increase of blood glucose level under hypercaloric conditions. Data expressed as mean ± standard deviation. (*): designates statistical significance in comparison with C group; (*p* < .05)

Monthly measurements of blood glucose levels of the control and the HD‐fed groups of *P. ob.* are shown in Figure 2. The animals were considered diabetic when blood glucose was >200 mg/dl. Our findings showed no significant variation in blood glucose levels throughout the experimentation in the control group. However, the animals subjected to HD showed a significant increase compared with controls (*p* < .05). Mean values increased from 119.75 ± 37.27 mg/dl at the start of the experimentation to 281.25 ± 95.18 mg/dl after 7 months and were classified as diabetic from the 3rd month (Baccouche et al., 2018; Dellaa et al., 2018).

After 3 months, HD‐induced hyperglycemia and obesity in *P. ob.* These results are in accordance with previous reports (Baccouche et al., 2018; Dellaa et al., 2018).

### 3.3 Evolution of erythrocyte aldose reductase activity in time

**Figure 3:**
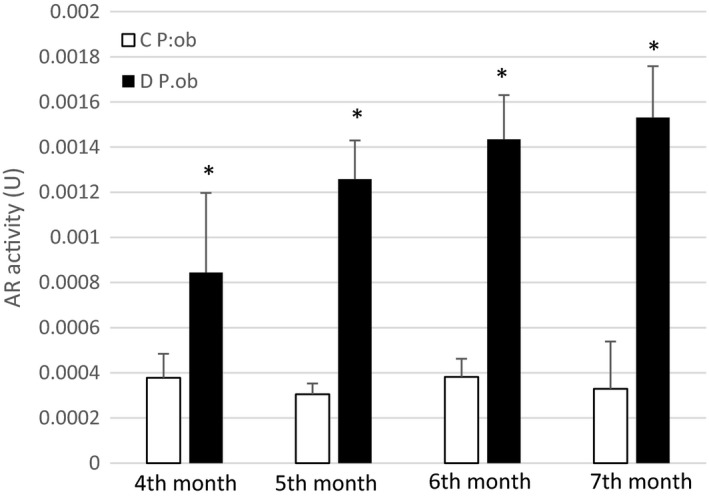
*In* *vivo* evolution of erythrocyte aldose reductase (AR) activity at different stages of hypercaloric diet in diabetic (D) *P. ob.* individuals (4th, 5th, 6th, and 7th month) versus *P. ob.* control (C). Data represent mean ± standard deviation of AR activity in D *P. ob.* (*n* = 4) and C *P. ob.* (*n* = 4). (*): designates statistical significance in comparison with Control group; (*p* < .05)

AR is a NADPH‐dependent enzyme. In vitro, it converts glyceraldehyde to glycerol with an equimolar oxidation of NADPH. Erythrocyte AR activity was measured in diabetics *P. ob*. from the 4th month of HD, which has been shown to be the earlier stage of the development of DR (Baccouche et al., 2017) to the 7th month (Saïdi, Mbarek, Chaouacha‐Chekir, et al., 2011; Saïdi, Mbarek, Omri, et al., 2011) and compared with *P. ob.* control AR activity. Our results (Figure 3) showed that AR activity increased progressively with longer diabetes duration but remained unchanged in control animals. Diabetic animals in the 4th, 5th, 6th, and 7th months showed a significant increase (*p* < .05) in AR activity compared with control animals ranging from 0.0008 U in the 4th month to 0.001 U in the 7th month in diabetic animals. Previous researches showed that the high activity of AR is caused by an increase of AR protein level (Liu et al., 2008; Nishimura et al., 1994) which is itself in correlation with the duration of diabetes and the prevalence of DR in diabetic patients (Oishi et al., 2002). The role of AR in the pathogenesis of diabetic complications is further supported by evidence that inhibition of the enzyme prevents and/ or delays the development of diabetic cataracts, neuropathy, nephropathy, and retinopathy (Hotta et al., 2006; Kawakubo, Mori, Sakamoto, Nakahara, & Ishii, 2012; Oates, 2010; Toyoda et al., 2014).

Our results showed that AR activity level could be a marker for the diagnosis of diabetes in *P. ob.,* which is, as demonstrated in our laboratory, an appropriate model to study diabetes and DR in humans (Dellaa et al., 2018, 2017; Saïdi, Mbarek, Chaouacha‐Chekir, et al., 2011; Saïdi, Mbarek, Omri, et al., 2011; Zhu, 2013).

### 3.4 Retina aldose reductase activity

**Figure 4:**
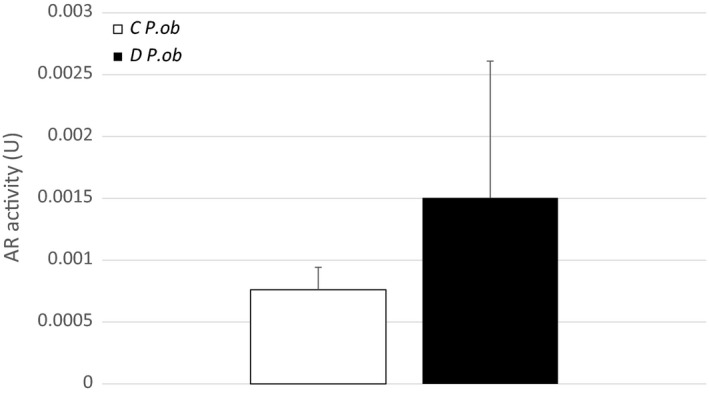
In vivo retinal aldose reductase (AR) activity in diabetic (D) and control (C) *P. ob.* at the 7th month of hypercaloric diet. Data represent mean ± standard deviation of AR activity in D *P. ob.* (*n* = 5) and C *P. ob.* (*n* = 2)

Preliminary measurements showed an increase in diabetic *P. ob.* retina AR activity (0.0015 U) compared with *P. ob.* control AR activity (0.0007 U) even though the difference is not statistically significant (Figure 4). The activation of the polyol pathway under hyperglycemic conditions results in the conversion of the glucose to sorbitol, which accumulates especially in pericytes leading to their degeneration and selective death. The loss of pericytes is the hallmark of DR in its early stages (Chung & Chung, 2005). However, at the 7th month of HD, the retinal metabolism of *P. ob.* may be only slightly affected. Therefore, a simple blood test could be helpful to measure the AR activity and to establish the diagnosis of the progress of the disease without sacrificing animals.

### 3.5 In vitro effect of astaxanthin on erythrocyte aldose reductase activity

**Figure 5:**
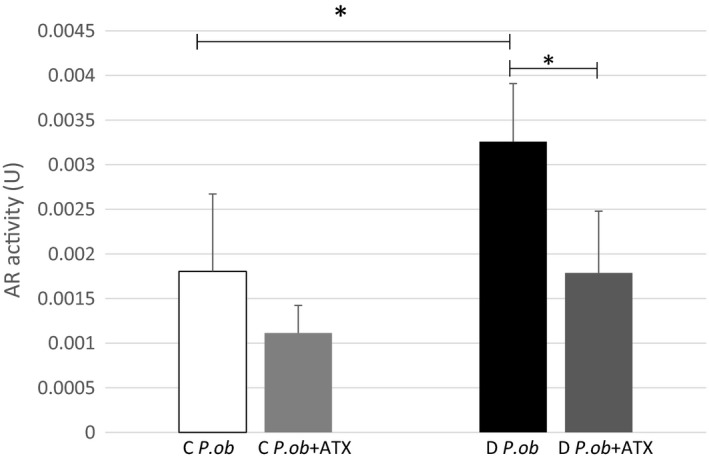
*In* *vitro* aldose reductase (AR) activity of control (C) (*n* = 4) and diabetic (D) *P. ob.* before (*n* = 4) and after (*n* = 4) adding astaxanthin (ATX). Data represent ± standard deviation. (*): designates statistical significance in comparison of D *P. ob.* before and after adding ATX; (*p* < .05)

Data show a decrease of the erythrocyte AR activity of *P. ob.* individuals after addition of ATX. In control animals, AR activity decreased slightly from 0.0018 to 0.0011 U, and the difference was not statistically significant (*p* > .05) (Figure 5). However, a significant decrease of AR activity was noticed in diabetic animals after addition of ATX (*p* < .05). Our results indicated that in vitro, ATX exhibited a significant direct inhibitory effect on erythrocyte AR activity probably by its particular structure via the polyene chain (the nonpolar part) binding to the hydrophobic site of AR slowing therefore its action.

AR has been a potential target for drug design, and its inhibition has been an attractive approach for the prevention and treatment of diabetic complications. Zenerestat, Zoporestat, quinoxalinone, sorbinil, and tolerstat (Zhu, 2013) are some of the synthetic inhibitors clinically tested whereas they have demonstrated a significant side effects. ATX is a naturally occurring pigment and has been proven to promote health condition (Yang et al., 2013).

### 3.6 In vivo effect of astaxanthin on erythrocyte aldose reductase activity

**Figure 6:**
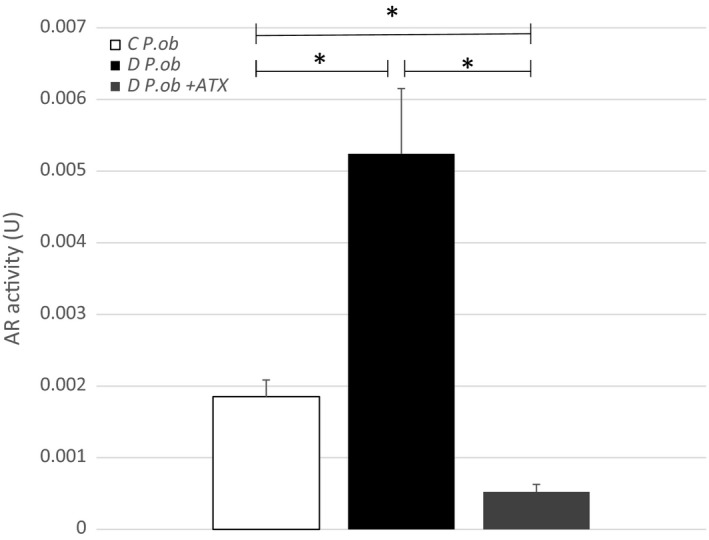
In vivo effect of astaxanthin (ATX) on erythrocyte aldose reductase (AR) activity in control (C) *P. ob.* (*n* = 4), in diabetic (D) *P. ob.* with hypercaloric diet during 7 months (*n* = 4) and in diabetic *P. ob*. fed with astaxanthin (ATX) the last week of the 7th month (*n* = 4). (*): designates statistical significance in comparison D *P. ob.* with *P. ob.* fed with ATX; (*p* < .05)

The results of the measurement of AR activity in vivo showed a significant decrease (*p* < .05) in diabetics *P. ob.* which received a daily dose of 4.8 mg/kg of ATX for a week starting from the 7th month of HD compared with control diabetics (Figure 6). These results suggest that ATX has a beneficial effect on DR through the inhibition of AR activity, which represents one of the major enzyme involved in the complications of diabetes and in particular of DR. It is possible to suggest that ATX could be an extremely interesting therapeutic candidate during the early stages of DR. Studies showed that ATX protected retinal cells cultured in high glucose levels by interfering with apoptosis mechanism and by preventing mitochondrial function from oxidative stress (Baccouche et al., 2017). A protective effect was confirmed in *vivo* with diabetic *P. ob*. fed with ATX for 1 week. It induced the antioxidant enzyme (HO‐1) and reduced glial reactivity (Baccouche et al., 2018). The powerful antioxydant effect of ATX could be explained by the several conjugated double bonds of the molecule which act by donating electrons and reacting with free radicals to convert them into a more stable product. Moreover, ATX has shown better biological activity than other antioxydants as it could bind to the cell membrane (Yuan, Peng, Yin, & Wang, 2011) which, due to its polyunsaturated fatty acid content and metabolic activities, endogenously generates free radicals and other oxydants (Hulbert, Pamplona, Buffenstein, & Buttemer, 2007).

## 4 CONCLUSION

The present study showed that erythrocyte AR activity of *P. ob*. increased in time with the evolution of diabetes and its main complication, DR. Therefore, AR activity might serve as an indicator for the onset and the progression of diabetes in this gerbil, which has been shown to develop similar structural and functional alterations to that observed for this disease in humans. ATX, a natural occurring carotenoid, is a potent inhibitor of AR activity both in vitro and in vivo and may prevent and treat diabetes complications in patients.

## CONFLICT OF INTEREST

The authors declare that there is no known conflict of interest associated with this work. Pr. Rafika Ben Chaouacha‐Chekir and Dr. Wissal Dhifi (PHD) contributed equitably to this work.

## ETHICAL APPROVAL

All applicable international, national, and/or institutional guidelines for the care and use of animals were followed.
